# Research on the Influence Mechanism of Rational Consumers’ Food Safety Supervision Satisfaction

**DOI:** 10.3390/ijerph16050739

**Published:** 2019-03-01

**Authors:** Jianhua Wang, Hanyu Diao, Lulu Tou

**Affiliations:** 1 School of Business, Jiangnan University, Wuxi 214122, China; 1080116222@vip.jiangnan.edu.cn (H.D.); 1080216411@vip.jiangnan.edu.cn (L.T.); 2 Jiangsu Research Center of Food Safety, Jiangnan University, Wuxi 214122, China

**Keywords:** consumption rationality, risk awareness, regulatory expectation, satisfaction towards food safety

## Abstract

As emerging food safety incidents have gained widespread concerns, research on consumers’ attitudes towards this issue is crucial to create effective solutions. To this end, in accordance with relevant data of consumers in 13 cities with subordinate districts, Jiangsu province, this paper divided different consumer groups by their experience so as to study their degree of satisfaction towards food safety and corresponding influencing factors. According to the descriptive statistics and the building of the cumulative logistic regression model, the results therefrom showed that consumers with direct or indirect experience have separate attitudes towards food safety which cannot be changed by changing consumers’ personal characteristics. Moreover, the two groups are divided in their demands in food production and consumption along with exceptions on policy implementation, etc. Finally, suggestions to improve consumers’ satisfaction are given in at the end of the paper.

## 1. Introduction

In recent years, many food safety incidents have occurred in China, such as the Melamine milk powder, Sudan red salted duck egg, sick dead pigs enter the market, and so on, which caused more than 50 thousand people to be sick or die. The whole society, especially consumers, are not optimistic about current food safety in China [1]. Such worries emerge as a major livelihood issue, which has negative impacts on social stability and government credibility [2]. Accordingly, the government has established relevant laws and regulations like the People’s Republic of China Food Safety Law to standardize the market order and require food safety quality. Though there have always been difficulties in supervision, the government is planning to perfect the Food Safety Law Implementation Regulations to standardize the behavior of law enforcement officers. In addition, the relevant government departments have clearly put forward “food safety strategies” targeting what is on our plates. To be more specific, measures include full implementation of food safety standards as well as improved information management and market inspection system, so as to promote a better market environment and overall situation. However, despite the achievements made in this field so far, the serious information asymmetry between producers and consumers, the lack of understanding and trust in the corresponding management and guarantee policies, and the government’s poor implementation of procedures [3,4] undermine consumers’ confidence in food safety. As a result, food safety is not equivalent to consumer satisfaction. Therefore, it is important to find out the reasons for the malcontent of consumers and ways to improve consumers’ satisfaction with food safety so that the information asymmetry can be decreased and the government’s public trust can be increased. All the aforementioned issues have become the focus of relevant departments and scholars.

As food production and consumption play a vital role in our daily life, food safety is closely linked with individuals directly or indirectly. In particular, experience may determine one’s awareness of food safety risks. Consequently, willingness to consume, consumption behavior, and satisfaction will then be affected.

Therefore, to explain the above issues, this paper analyzes real opinions of consumers with different experiences then explores the influencing factors that can have an effect on consumers’ satisfaction. In addition, some related suggestions can be made to upgrade production and consumption processes, further understanding of consumers themselves, and promotion of supporting policies together with improved government supervision. The ultimate goal of the paper is to keep abreast of various needs and thus to facilitate the government role in this setting, correct ideas about food safety, and improve consumers’ satisfaction.

In summary, this paper has divided all consumers into two groups according to their experience to study factors that have potential influence on consumers’ opinions so as to propose relevant suggestions in response to different needs and inadequate supervision of the government, which are the main contributions to the existing literature.

## 2. Literature Review and Hypotheses

### 2.1. Literature Review

Food security means an adequate and safe food supply [5]. According to the revised Food Safety Law in 2015, the definition is nontoxic and harmless food that meets nutritional requirements and does not cause acute, sub-acute or chronic harm to human health [6]. With regard to public satisfaction, opinions vary in line with their respective research. Some believe that the public will evaluate products and services according to personal needs, expectations, and goals. The consequent feelings should be equal to consumers’ degree of satisfaction [7]. Since the 1970s, some developed countries in the West have adopted the concept to assess government services. For instance, the American customer satisfaction index (ACSI) proposed by the National Quality Research Center and the American Society for Quality was a typical economic index based on a performance evaluation system [8]. Considering our national conditions and social characteristics, scholars adjusted variables appropriately in accordance with the ACSI model to put forward an index system in China’s setting, which is applied in government departments [9,10], railway service [11], customer reactions [8], etc. Judging from the above definition and application, the major perspective is the external objective environment and consumers’ subjective perception.

In terms of the external objective environment, surveys have been conducted to mainly investigate the impact of population characteristics, hazardous substances, governance along with supervision, and so on. In addition, comparisons of certain regions were also made. Smith and Dominic et al. [1] compared the public’s concern, understanding, and trust in food safety in Japan and Australia. What is more, Choi et al. [12] analyzed the public satisfaction with understanding, attitude, and standards on food safety. Research based on Hungarian consumers showed that awareness of food safety risks is divided by groups, education, and social strata [13]; especially the elderly and women were more concerned with this issue. Moreover, da Cruz et al. [14] pointed out that consumers were also subject to the expertise, transparency, honesty, and goodwill of regulatory authorities apart from label information [15]. Furthermore, Sayin [16] claimed that influence from friends, families, colleagues, and public officials was also a factor that can influence consumers’ satisfaction with food safety. Through research on consumers in Japan, Canada, and the United States, it was found that satisfaction with food safety and personal direct experience were closely correlated; the same was true for Chinese domestic consumers [17].

However, from consumers’ subjective perception, consumption confidence as well as situations and factors of such ideas are the main focus [18,19]. Consumers’ risk awareness can be defined as their evaluation of all the potential risks [20], which is conducive to clarifying people’s attitude to risk and perceptual judgment [21]. Such subjective judgments or deductions about uncertainty or unfavorable results [22] can reflect value, superficial characteristics, history, and ideology far beyond individuals [23]. Specifically, this social and cultural concept refers to consumers’ description of certain situations, risk control, probability expectation, and confidence in such estimates [20]. The Fishbein model (multi-attribute model) [24] allows attitude to determine result evaluation and behavioral prediction due to behavioral intention. Taking health, psychology, money, time, and quality as measurement items, Ruth et al. [25] found that dimensions of risk perception mainly include “consequence seriousness”, “safety concern”, “unconsciousness of risk exposure”, “environmental harm”, “information”, “risk exaggeration”, and “moderate adjustment”. In light of consumers’ quality judgement, products could be divided into search, experience, and trust types [26]. That is to say, for some products, it was hard to tell whether they were of good quality or not ahead of purchase or even after use. In that way, belief or psychological, cultural, and social factors should be taken into consideration apart from the potential health risk [5]. By adopting the psychological measurement paradigm of the Slavic risk characteristic assessment scale [27], Elise [28] carried out studies on participants’ judgment, importance ranking, correlation analysis, and factor analysis, so as to obtain perception data and corresponding factors. Moreover, a map of risk perception was finished, which indicated that familiarity, uncontrollability, and gravity were decisive. In conclusion, food safety issues with high concern but lower satisfaction should be serious and widespread ones that the public are not familiar with. In addition, Frewer et al. [29] considered panic as an important factor, namely, the fear of serious consequences owing to food safety incidents. Another factor of significance was familiarity, as lack of knowledge would result in much more worries. As a result, subjective perception rather than objective risks determines consumption behavior [30]. Meanwhile, inadequate effective technologies made it an issue of trust [31]. Different levels of risk perception regulate trust and willingness, which in turn further affects final attitude [32]. To be precise, trust is the sure expectation that the other party will certainly fulfill their duties in social interactions [33]. In other words, accuracy, knowledge, and concern for public welfare constitute trust [29]. From the perspective of new institutional economics [29], emphasis on trust from the level of economic behavior, the generation of trust in economic activities is due to the continuous interpersonal relationship and social network. On this basis, some scholars believe that such trust is the customer’s view of credibility and friendliness of salesmen. If consumers trust salesmen, they will believe the other party will do their part well [34] and do no harm at least [35]. Such trust is the comprehensive outcome of the ability to be counted on, credit, reliability, integrity, goodwill, and information provision [5].

Others also analyzed reasons of lower satisfaction in the view of supply and demand [36,37,38]. Quality is a crucial property of food safety. However, market failure prevails due to inadequate and unequal information, which therefore fails optimal supply. In that way, consumers cannot choose totally in accordance with preference and budget and products are not fully utilized, thus leading to a widespread unsatisfied feeling [36,37,38].

In summary, scholars have carried out an overall analysis of consumers’ view and influencing factors. However, their studies can merely reflect the basic situations instead of precise positioning to the group fueled by discontent. According to whether the consumer has experienced food safety incidents personally, this paper has divided them into consumers with direct experience and consumers with indirect experience. Hence, this paper discusses the role of experience in affecting consumers’ attitude and relevant factors based on previous research, so as to provide corresponding suggestions for improving satisfaction with food safety.

### 2.2. Consumers’ Experience and Attitude to Food Safety

#### 2.1.1. Consumers’ Experience and Attitude to Food Safety

Though in low probability, consumers are relatively vulnerable to food safety incidents [39]. According to Wen et al. [40], people who are active in a particular social group always play down some risks and emphasize others, in order to maintain and control this group. Therefore, consumers’ real concern is the severity of incidents. That is to say, if such incidents happen, the results will decide people’s ideas of risk [38], then their degree of satisfaction. In most cases, “rule of thumb” is the main principle for consumers’ judgments. Nevertheless, the fact is that consumers believe similar cases will happen, so that they will turn to familiar models regardless of reasons or likeliness to repeat. In particular, consumers with direct relevant experience are more likely to overestimate the severity of final outcomes [30]. Let us suppose:

**Hypotheses** **1.** *Degree of satisfaction differs among consumers with direct experience and those with indirect experience.*

**Hypotheses** **1a.** *Consumers with direct experience are less satisfied with food safety.*

**Hypotheses** **1b.** *Consumers only with indirect experience are more satisfied with food safety.*

#### 2.2.2. Consumers’ Experience and Policy Influence

The policy influence refers to the effectiveness of the policy measures implemented by the relevant regulatory authorities on food safety issues. Mockshell et al. [41] argued that considering irrational and illogical characteristics in consumers’ behaviors, their awareness of risk was actually divergent from that of policy makers’. For specific information aimed at eliminating food safety uncertainties, consumers either cannot obtain, or will not recognize and process it. To be noted, consumers’ views of risk and degree of satisfaction comprise two stages: Information processing and feedback. However, anchoring and adjustment heuristics exists in the former stage [30], which means that owing to the lack of timely and enough government supervision, food safety will not totally change people’s established idea of food safety risks if such incidents happen. Moreover, the latter stage has witnessed loss aversion and confirmation bias. In view of loss aversion, satisfaction feelings will not improve with most guarantees but will decrease sharply with few serious incidents. Then, the latter factor will further consolidate “stereotypes” to confirm food safety through negative information. Here are the relevant hypotheses:

**Hypotheses** **2.** *Policies related to food safety management have different influence on consumers with direct and indirect experience.*

**Hypotheses** **2a.** *Policies on food safety management have less influence on consumers with direct experience.*

**Hypotheses** **2b.** *Policies on food safety management have greater influence on consumers with only indirect experience.*

#### 2.2.3. Consumers’ Experience and Regulatory Expectations

Regulatory expectations mean public hope for government supervision in terms of food safety. Generally, domestic consumers are not optimistic about food safety in China. For those “experience products”, consumers will judge after personal experience, but actually, reasonable analysis makes sense only under certain emotions [42]. According to the hypothesis of “risk from emotion”, the interaction of emotions and evaluation determines individual behaviors. In addition, emotions can buffer against the effect of evaluation on action [43]. Once food safety incidents occur, consumers will have negative emotions, which will greatly affect their attitude towards risks and regulatory efforts. The “maximum social risk” theory [44] says that the greatest loss of risks will make people tend to neglect its probability. Similarly, indirect negative influence of food safety incident will outweigh the corresponding direct harm [1]. In that way, regulatory expectations will go against the public view of satisfaction and risk perception, namely, consumers’ regulatory expectations exceed adverse aspects of uncertainty and lead their view in these two aspects. Conversely, consumers’ view will also have an impact on expectations of supervision. Supposing:

**Hypotheses** **3.** *Consumers with direct experience and those with only indirect experience have different expectations from government regulation.*

**Hypotheses** **3a.** *Consumers with direct experience have fewer expectations from government regulation.*

**Hypotheses** **3b.** *Consumers with only indirect experience expect more from government regulation.*

## 3. Data Source and Sample Feature Analysis

### 3.1. Data Source

**Figure 1:**
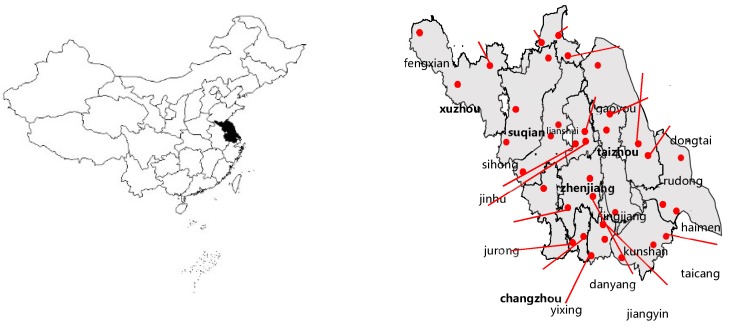
Research areas.

Research was conducted at large and medium-sized supermarkets, shopping malls, large-scale farmers’ markets, rural markets, along with other urban and rural gathering sites of consumers in 13 cities with subordinate districts, Jiangsu Province, East China, from December in 2017 to February in 2018. Data were collected from local consumers who worked or lived for more than half a year, so as to gain a basic understanding of consumers’ degree of satisfaction towards food safety in China. Following the principle of specific designs of different targets, the research selected typical towns and districts which had high, middle, and low GDP in 2016 (see Table 1 and Figure 1 below). The questionnaire consisted of 32 questions divided into 5 parts about personal statements, awareness of food safety, status assessment of food safety, evaluation of food supervision work, overall evaluation of food safety satisfaction, and suggestions for food safety work, respectively. The major method included questionnaires through random sampling and face-to-face interviews of about 20–30 minutes down by trained personnel (generally, there were 10–20 trained people to conduct the interview in every place, and the specific quantity depended on the place), which covered local residents of different gender, age, education, and vocation ranging from 18 to 65 years old. Another sampling process was then conducted in proportion to groups of gender, urban or rural, as well as registered or nonregistered residents. In order to minimize data errors, surveyors first explained the previously related terms. Finally, a total of 5511 questionnaires were sent out and to ensure a certain amount and the quality of the questionnaires; the investigators and respondents were paid according to the questionnaires they submitted. After excluding invalid responses, 5131 questionnaires were collected, so the validity was 93.11%.

### 3.2. Descriptive Statistics

After preliminary pre-analysis, the consumers involved were divided into two groups: One referred to those with direct experience of food safety incidents; the other comprised those with indirect experience. The basic characteristics of consumers in different groups are shown in Table 2 after descriptive statistical analysis.

Among the 5131 interviewees, consumers with indirect experience accounted for 58.8%. As sampling was conducted at random, the figure shows that in daily life, most consumers have no direct experience. Moreover, there were 53.5% female interviewees with indirect experience with food safety incidents, which is higher than their male interviewee counterparts, 46.5%. In terms of age structure, interviewees were mainly from 20 to 50 years old as 73.1% of those with direct experience, and 73.2% of the rest were within this age range. In addition, interviewees with direct experience and the rest shared a similar education background; specifically, the ratios of those with college or higher degrees were 53.3% and 53.6%, respectively, in the two groups. The lower the respondents’ education level was, the less experience they had. As for their vocations, more experienced interviewees were mainly civil servants and freelancers, accounting for 44.6% and 31.4%, respectively, among those with direct experience as well as 44.8% and 28.3% separately among other interviewees. In particular, the majority of interviewees were registered local residents in Jiangsu province and urban areas, accounting for 90.8% and 58.1% separately among those with direct experience as well as 90.5% and 57.7%, respectively, among those with indirect experience.

### 3.3. Analysis of the Satisfaction with Food Safety of Consumers in Different Types 

In line with relevant documents and standard files of customers’ degree of satisfaction, this paper set five grades, namely, very satisfied, relatively satisfied, general, not that satisfied, and unsatisfied. Furthermore, a Likert scale was adopted to quantify collected data, as shown in Table 3 below.

From Table 2, comparing extreme ratings of “very satisfied” and “not satisfied”, the remaining three grades accounted for a larger share. To be specific, 82.2% of interviewees with direct experience and 88.1% of those with indirect experience chose grade “general” or above. Based on this, the conclusion can be drawn that though they held different views, consumers with direct experience prefer lower rates of food safety government regulation satisfaction. Thus, Hypotheses 1, 1a, and 1b are true in this sense.

## 4. Model Estimation and Discussion

### 4.1. Model Building

If variables are in ordinal scale and not estimated by the general linear, logistic regressions in the number of fitting dependent variable minus 1 can be adopted to form a cumulative logistic regression model. In order to better study the different categories of consumers and the factors affecting food safety satisfaction, we used the models above as a tool.

Taking the five grades in this paper as an example, assume that the values of dependent variables are 1, 2, 3, 4, and 5 separately. Accordingly, four fitted models of 12 selected dependent variables are as follows, considering the probabilities of value levels is *π*_1_, *π*_2_, *π*_3_, *_π_*_4,_ and *π*_5_:

(1) $$\mathrm{logit}\frac{\pi_{1}}{1-\pi_{1}}=\mathrm{logit}\frac{\pi_{1}}{\pi_{2}+\pi_{3}+\pi_{4}+\pi_{5}}=-\alpha_{1}+\beta_{1}x_{1}+\cdots +\beta_{12}x_{12}$$

(2) $$\mathrm{logit}\frac{\pi_{1}+\pi_{2}}{1-\left(\pi_{1}+\pi_{2}\right)}=\mathrm{logit}\frac{\pi_{1}+\pi_{2}}{\pi_{3}+\pi_{4}+\pi_{5}}=-\alpha_{2}+\beta_{1}x_{1}+\cdots +\beta_{12}x_{12}$$

(3) $$\mathrm{logit}\frac{\pi_{1}+\pi_{2}+\pi_{3}}{1-\left(\pi_{1}+\pi_{2}+\pi_{3}\right)}=\mathrm{logit}\frac{\pi_{1}+\pi_{2}+\pi_{3}}{\pi_{4}+\pi_{5}}=-\alpha_{3}+\beta_{1}x_{1}+\cdots +\beta_{12}x_{12}$$

(4) $$\mathrm{logit}\frac{\pi_{1}+\pi_{2}+\pi_{3}+\pi_{4}}{1-\left(\pi_{1}+\pi_{2}+\pi_{3}+\pi_{4}\right)}=\mathrm{logit}\frac{\pi_{1}+\pi_{2}+\pi_{3}+\pi_{4}}{\pi_{5}}=-\alpha_{4}+\beta_{1}x_{1}+\cdots +\beta_{12}x_{12}$$

Compared to the traditional two-category logistic regressions, values counted by logistic operation are *π*_1_, *π*_1_ + *π*_2_, *π*_1_ + *π*_2_ + *π*_3_, and *π*_1_ + *π*_2_ + *π*_3_ + *π*_4_, respectively, in other words, cumulative probabilities of dependent variables’ values in order. The goal is to expand the value range to (−∞, +∞).

From the above, this model actually divides the dependent variables into two levels according to different values and establishes a logistic regression model with two categories. Regardless of the beak point in the model, the coefficients *β*i of each variable remain unchanged, and only the constant term alpha is changeable. Thus, the OR value is the ratio between rise of dependent variables with each unit change of the dependent variables. For the aforementioned three models, the respective partial regression coefficient is constant, which is one of the prerequisites for fitting the cumulative logistic model.

In particular, differing from two-category logistic regression, the fitted models in this paper should use “minus sign” rather than “plus sign” ahead of the constant term. The reason is that the constant term here is in line with the situation that the lower level compares to the higher level and has opposite meaning with traditional ones apart from the order of *α*_1_ < *α*_2_ < *α*_3_ < *α*_4_. As the main focus is *β*i, the results will not be greatly divergent.

*π*_1_, *π*_1_ + *π*_2_, *π*_1_ + *π*_2_ + *π*_3_, and *π*_1_ + *π*_2_ + *π*_3_ + *π*_4_ can be calculated according to Equations (1), (2), (3), and (4) separately, then *π*_5_ can be figured out through equation *π*_1_ + *π*_2_ + *π*_3_ + *π*_4_ + *π*_5_ = 1.

(5) $$\pi_{1}=\frac{\exp\left(-\alpha_{1}+\beta_{1}x_{1}+\cdots +\beta_{12}x_{12}\right)}{1+\exp\left(-\alpha_{1}+\beta_{1}x_{1}+\cdots +\beta_{12}x_{12}\right)}$$

(6) $$\pi_{2}=\frac{\exp\left(-\alpha_{2}+\beta_{1}x_{1}+\cdots +\beta_{12}x_{12}\right)}{1+\exp\left(-\alpha_{2}+\beta_{1}x_{1}+\cdots +\beta_{12}x_{12}\right)}-\pi_{1}$$

(7) $$\pi_{3}=\frac{\exp\left(-\alpha_{3}+\beta_{1}x_{1}+\cdots +\beta_{12}x_{12}\right)}{1+\exp\left(-\alpha_{3}+\beta_{1}x_{1}+\cdots +\beta_{12}x_{12}\right)}-\pi_{1}-\pi_{2}$$

(8) $$\pi_{4}=\frac{\exp\left(-\alpha_{4}+\beta_{1}x_{1}+\cdots +\beta_{12}x_{12}\right)}{1+\exp\left(-\alpha_{4}+\beta_{1}x_{1}+\cdots +\beta_{12}x_{12}\right)}-\pi_{1}-\pi_{2}-\pi_{3}$$

(9) $$\pi_{5}=1-\pi_{1}-\pi_{2}-\pi_{3}-\pi_{4}$$

### 4.2. Description about Variables

According to our questionnaire, the food safety status assessment section consisted of daily food like rice and flour, edible oil, meat and meat products, vegetable and fruit, dairy products, aquatic products, eggs, etc., and some ordinary places where we buy food like the supermarket, convenience store, chain food store, etc. (We gave all of these examples in our questionnaires to help the respondents to understand the question and make the right choices.) To find out whether daily food and ordinary markets are factors, we only needed to choose one of the examples we gave in our questionnaires to represent all the things. After analyzing the data, we found that all the things above have a similar effect on our results, so we chose one of the most typical options in the main text and explained why we chose them. When it came to the food safety supervision work evaluation section and overall evaluation of food safety satisfaction, variables X4 to X12 were only the raw data from the original questionnaire. Thus, no generalizations can be made. Table 4 shows variables’ meanings, values, and corresponding statistics. Here are the illustrations of variables’ values plus partial variables’ meanings:

Value assignment. The five grades mentioned above correspond to five figures. Specifically, 1 equals “unsatisfied/ highly worried”, 2 “not that satisfied/ worried”, 3 “general”, 4 “relatively satisfied/ not that worried”, and 5 “very satisfied/ not worried”.

Aquatic products. Consumers believe that greater potential danger exists in aquatic products that are demanding in freshness, vulnerable to contamination, and hard to test. In addition, products involved in the questionnaire included rice, flour meat and meat products, dairy products, eggs, beverages, edible oils, vegetables and fruits, soybeans and nuts, and snacks and non-staple foods. Statistical analysis of the attained data on people’s attitudes supports the conclusion that aquatic products rank in the middle among the above categories. No extremes are seen accordingly, which means the results gained are the most ideal.

Farmers’ markets. The questionnaire also asked consumers’ attitudes towards farmers’ markets, including large supermarkets, convenience stores, catering chains, stalls, rural fairs/morning and night markets, online ordering, regional food manufacturers, and so on. The final outcomes after analysis suggest that farmers’ markets show greatest similarity with degree of satisfaction. In addition, well-distributed figures also indicate ideal results. As this research targeted both consumers in small towns and cities, common farmers’ markets featuring moderate prices and a full range of products are attractive to consumers. Therefore, they can serve as a good representation of trade places of food.

### 4.3. Results and Analysis

The ordered logistic regression method was adopted to evaluate the degree of satisfaction. Clearly, the two groups of consumers have varied opinions of food safety satisfaction. In addition, different factors have different effects on different consumers’ satisfaction with food safety. The specific analysis is as follows.

The result is *p* < 0.001 through the likelihood-ratio test (whether all partial regression coefficients of independent variables are 0), indicating that independent variables have a better goodness of fit.

In addition, the analysis of consumers’ individual characteristics found that the two groups demonstrated similar features and only the gender factor can significantly change consumers’ degree of satisfaction, which means that men are more concerned about the food safety issue for both consumer groups, while other personal characteristics do not influence the evaluation of the satisfaction degree of food safety. Maybe the result here is inconsistent with the above analysis; for example, there are more men in the consumer group with direct experience. Though this cannot be explained in this paper, further study about it is planned.

With regard to Table 5 the results showed that different policies have varied influence on consumers. Firstly, it shows that regulators’ efforts to crack down on illegal activities have the same effect on consumers with only direct or indirect experience, which means that regulators’ efforts to crack down on illegal activities will affect all kinds of consumers’ satisfaction with food safety in the same way. Apart from that, though through increasing the satisfaction with information disclosure of penalty information by regulatory authorities, overall satisfaction is increasing, this is more useful to consumers with direct experience. For them, inadequate satisfaction with information disclosure will be greatly influential and lead to much worse comments on food safety, with more severe influence than that on on those with only indirect experience. With regard to implemented regulatory measures, such as publicity and education, regular supervision and evaluation, regular sampling and information transparency, they have greater influence on consumers with only indirect experience than those with direct experience. Similarity, if the latter group is not satisfied with the implementation results, their attitudes will change accordingly. However, their change is much less than that of the former group, which may be caused by different levels of trust in the government. Therefore, H2, H2a, and H2b are correct. The change may be caused by different levels of trust in the government.

As shown in Table 6, with the increase in satisfaction with food safety supervision, overall satisfaction is also increasing for all kinds of consumers, which means that the lower the satisfaction with food safety supervision, the worse the expectations of it, and worse regulatory expectations from consumers can equal a basically lower degree of satisfaction. Moreover, when the degree of food safety supervision satisfaction increases by one level, it has a greater impact on consumers with indirect experience. As a result, the idea is more applicable to consumers with only indirect experience instead of those with direct experience. Thus, these two groups have different expectations of government actions, and the former group have higher expectations. This is probably caused by the government having a lower credibility in direct-consumers’ minds. In that way, H3, H3a, and H3b have then been verified.

## 5. Discussion

Based on the sample data of 5131 consumers in 13 cities with subordinate districts in Jiangsu province, this paper conducted research on the degree of satisfaction and needs concerning food safety for two consumer groups, those with direct experience and others with indirect experience, and then further analyzed their satisfaction and its influencing factors. 

First, the findings reveal that we cannot change consumers’ food safety satisfaction by changing their personal characteristics. Bánátiand Lakner [45] found that consumers of different literacy levels and social classes have different perceptions of food safety risks; this was based on Hungarian consumers, and it is different from our findings in China, which may be due to cultural factors and daily habits.

Secondly, the results showed that consumers with different experience types have varied degrees of satisfaction towards food safety, which is the same conclusion as that reached by Tonsor and other researchers. Psychological panic and familiarity are often important factors influencing food safety risk perception, which in turn affects food safety satisfaction. Because consumers with direct experience knew more about the food safety risk than the other kind of consumers, they were more familiar with that, and their psychological panic may be more reasonable and rational. In addition, the experience can also help them to learn more about the government’s regulation of food safety; as a result, the government may have a better or worse credibility in their minds, which can directly influence the confidence to and expectations from the government. Therefore, based on the findings above and the Fishbein model (multi-attribute model) [25], the study found that information of food safety disclosure, relevant knowledge, and government measures all have a different impact on different kinds of consumers, which is the same as Peter’s research.

This is an innovation point of this paper. However, there are still some shortcomings and limitations, such as that the data processing method is relatively simple, and the reasons for the conclusion are not deeply explored. In the future, the data processing method will be changed, and the deep influencing factors of satisfaction and the above conclusions will be further analyzed.

## 6. Conclusions

On the one hand, the results show that personal characteristics do not have a significant impact on all kinds of consumers, and people with different characteristics probably have the same food safety satisfaction. On the other hand, with regard to consumers’ supervision evaluation and regulatory satisfaction, the results showed that consumers with indirect experience pay more attention to their food safety knowledge, and consumers with direct experience may consider food safety information more important. As a result, to improve the food safety satisfaction of consumers with indirect experience, it is advisable for the government to establish a long-term organized education system to enhance consumers’ safety awareness, cultivate good habits, build their confidence in consumption, and standardize consumption behaviors. Further, to increase direct-experienced consumers’ food safety satisfaction, a big data platform of food safety based on the Internet of Things and traceability system of food information should be a priority to conduct risk assessment, real-time monitoring, and dynamic reviews. In that case, innovation can further upgrade the risk management of food safety so that real information on food safety is available to the public and the market witnesses less negative influence of information asymmetry, moral risks, and adverse selection in food market. Apart from that, in order to minimize food safety risks and improve people’s food safety satisfaction, restrict relevant parties’ behaviors, as well as define different parties’ responsibilities, profits, and duties, joint efforts by multiple players in society should be made. What is more, the transmission of food safety information can be more efficient without direct interference in the market from the supervision side.

## Figures and Tables

**Table 1:** Comparative statistics of personal characteristics of sample respondents with or without direct experience.

Social Demographic Characteristics	Characteristic	Sample Size		Percentage(%)	
		Indirect Experience	Direct Experience	Indirect Experience	Direct Experience
Age	<20	271	173	9.0%	8.2%
	20–30	865	569	28.7%	26.9%
	31–40	721	527	23.9%	25.0%
	41–50	623	449	20.6%	21.2%
	51–60	373	286	12.3%	13.5%
	>60	165	109	5.5%	5.1%
Sex	Male	1405	1056	46.5%	50%
	Female	1613	1057	53.5%	50%
Highest Degree	Colleague degree or above	1614	1126	53.6%	53.3%
	High school and technical secondary school	756	520	25.1%	24.6%
	Junior High School	443	337	14.6%	15.9%
	Primary School or Below	205	130	6.8%	6.2%
place of domicile	Native	2177	1557	72.1%	73.7%
	Other cities in Jiangsu	556	361	18.4%	17.1%
	Outside of Jiangsu	285	195	9.5%	9.2%
household registration type	City (town) residents	1742	1228	57.7%	58.1%
	Rural Household	1276	885	42.3%	41.9%
Jobs	Government Employee	1351	943	44.8%	44.6%
	Freelancer	855	663	28.3%	31.4%
	Farmer	223	131	7.4%	6.2%
	Students	368	235	12.2%	11.1%
	Retirees and unemployed people	221	141	7.3%	6.7%

**Table 2:** A general description of the satisfaction evaluation of government regulation of food safety with or without direct experience.

Satisfaction Rating	Direct Experience		Indirect Experience	
	Population	Percentage	Population	Percentage
Very satisfied	151	7.2%	280	9.3%
Satisfied	631	29.8%	1071	35.5%
General	956	45.2%	1307	43.3%
Not so satisfied	282	13.3%	307	10.2%
Not satisfied	93	4.4%	53	1.7%
total	2113	100%	3018	100%

**Table 3:** Variables’ meanings, values, and corresponding statistics.

Type	Variables	Variables Values	Mean Value		SD	
			Direct Experience	Indirect Experience	Direct Experience	Indirect Experience
Individual Characters	**Age**	<20 = 1; 20–30 = 2; 31–40 = 3; 41–50 = 4; 51–60 = 5; >60 = 6	3.204	3.152	1.331	1.346
	Sex	Male = 1; Female = 2	1.5	1.535	0.5	0.499
	Degree	Primary School and below = 1; Junior High School = 2; High school and technical secondary school = 3; Collage or above = 4	2.654	2.659	1.157	1.147
	household registration type	urban registration = 1; Rural household = 2	1.417	1.423	0.493	0.494
Food safety status assessment	Aquatic products (X1)	Worry a lot = 1; Worry a bit = 2; General = 3; Not very worry about = 4; Never worry about = 5	2.843	2.768	1.366	1.396
	Farmer Market (X2)	Worry a lot = 1; Worry a bit = 2; General = 3; Not very worry about = 4; Never worry about = 5	2.896	2.797	1.23	1.22
Food safety status assessment	Catering enterprise health environment (X3)	Dissatisfied = 1; not quite satisfied = 2; General = 3; Satisfied = 4; Very Satisfied = 5	2.762	2.587	0.999	0.964
Food safety supervision work evaluation	Publicity and education work (X4)	Dissatisfied = 1; not quite satisfied = 2; General = 3; Satisfied = 4; Very Satisfied = 5	2.801	2.632	0.915	0.842
	Daily regulatory assessment (X5)	Dissatisfied = 1; not quite satisfied = 2; General = 3; Satisfied = 4; Very Satisfied = 5	2.771	2.619	0.894	0.848
	Daily sampling (X6)	Dissatisfied = 1; not quite satisfied = 2; General = 3; Satisfied = 4; Very Satisfied = 5	2.804	2.633	0.914	0.865
	Food safety information disclosure (X7)	Dissatisfied = 1; not quite satisfied = 2; General = 3; Satisfied = 4; Very Satisfied = 5	2.861	2.697	0.947	0.89
Food safety supervision work evaluation	Fight against illegal activities (X8)	No efforts = 1; Minor efforts = 2; General = 3; A bit effort = 4; very powerful = 5	3.003	2.802	0.969	0.919
Food safety supervision work evaluation	Penalty information disclosure (X9)	Dissatisfied = 1; not quite satisfied = 2; General = 3; Satisfied = 4; Very Satisfied = 5	2.917	2.748	0.967	0.906
Overall evaluation of food safety satisfaction	Knowledge of food safety (X10)	Dissatisfied = 1; not quite satisfied = 2; General = 3; Satisfied = 4; Very Satisfied = 5	2.724	2.62	0.874	0.827
	Local food confidence level (X11)	Worry a lot = 1; Worry a bit = 2; General = 3; Not very worry about = 4; Never worry about = 5	2.651	2.483	0.836	0.806
	Overall satisfaction with the status (X12)	Dissatisfied = 1; not quite satisfied = 2; General = 3; Satisfied = 4; Very Satisfied = 5	2.656	2.511	0.812	0.798

**Table 4:** Two types of consumers’ personal characteristic model parameter estimation results.

Variables	Refer to	*β*		Wald		*p* value		OR value	
		Direct Experience	Indirect Experience	Direct Experience	Indirect Experience	Direct Experience	Indirect Experience	Direct Experience	Indirect Experience
age	>60							1.000	1.000
<20		0.261	0.245	0.560	1.110	0.454	0.292	1.245	1.277
20–30		0.240	0.197	0.703	1.068	0.402	0.301	1.218	1.217
31–40		0.206	0.209	0.501	1.256	0.479	0.262	1.177	1.232
41–50		−0.093	−0.120	0.339	0.432	0.560	0.511	0.876	0.887
51–60		0.008	0.107	0.010	0.353	0.921	0.553	0.978	1.113
sex	Male							1.000	1.000
female		−0.217	−0.169	6.990	5.914	0.008	0.015	0.802	0.845
degree	Colleague or above							1.000	1.000
High school and technical secondary school		0.047	0.085	0.089	0.432	0.766	0.511	1.048	1.089
Junior high school		−0.013	0.028	0.009	0.068	0.923	0.794	0.988	1.028
Primary school or below		−0.003	0.014	0.000	0.020	0.983	0.887	0.997	1.014
household registration type	Rural household							1.000	1.000
urban registration		0.098	−0.048	1.068	0.393	0.301	0.531	1.103	0.953

**Table 5:** Two types of consumer supervision evaluation model parameter estimation results.

variables	Ref to	*β*		Wald		*p* Value		OR Value	
		Direct Experience	Indirect Experience	Direct Experience	Indirect Experience	Direct Experience	Indirect Experience	Direct Experience	Indirect Experience
Publicity and education work	Very satisfied							1.000	1.000
Very dissatisfied		−1.193	−0.771	13.699	6.436	0.000	0.011	0.303	0.462
Not very satisfied		−1.001	−0.774	13.429	12.042	0.000	0.001	0.367	0.461
general		−0.680	−0.583	7.719	9.675	0.005	0.002	0.506	0.558
satisfied		−0.297	−0.362	1.596	4.251	0.206	0.039	0.743	0.696
Daily supervision evaluation	Very satisfied							1.000	1.000
Very dissatisfied		−1.616	−1.368	20.746	16.425	0.000	0.000	0.199	0.255
Not very satisfied		−0.897	−0.765	10.211	10.542	0.001	0.001	0.408	0.465
General		−0.551	−0.418	4.980	4.502	0.026	0.034	0.577	0.658
Satisfied		−0.135	0.002	0.333	0.000	0.564	0.991	0.874	1.002
Daily sampling	Very satisfied							1.000	1.000
Very dissatisfied		−2.322	−1.598	39.583	20.504	0.000	0.000	0.098	0.202
Not very satisfied		−1.779	−1.465	34.438	34.986	0.000	0.000	0.169	0.231
General		−1.381	−0.883	25.621	16.643	0.000	0.000	0.251	0.414
Satisfied		−0.817	−0.589	10.309	8.740	0.001	0.003	0.442	0.555
Food safety informationdisclosure	Very satisfied							1.000	1.000
Very dissatisfied		−1.226	−1.237	12.285	13.557	0.000	0.000	0.294	0.290
Not very satisfied		−1.292	−1.051	18.440	17.923	0.000	0.000	0.275	0.350
General		−1.013	−0.854	13.762	15.091	0.000	0.000	0.363	0.426
Satisfied		−0.732	−0.458	8.042	5.014	0.005	0.025	0.481	0.633
Fight against illegal activities	Very satisfied							1.000	1.000
Very dissatisfied		−1.453	−1.455	19.448	22.523	0.000	0.000	0.234	0.233
Not very satisfied		−1.277	−1.517	23.486	46.653	0.000	0.000	0.279	0.219
General		−0.998	−0.990	16.527	24.070	0.000	0.000	0.369	0.372
Satisfied		−0.587	−0.533	6.138	7.968	0.013	0.005	0.556	0.587
Penalty information disclosure	Very satisfied							1.000	1.000
Very dissatisfied		−2.470	−2.553	49.903	59.407	0.000	0.000	0.085	0.078
Not very satisfied		−1.749	−1.733	33.604	48.838	0.000	0.000	0.174	0.177
General		−1.131	−1.167	16.708	26.826	0.000	0.000	0.323	0.311
Satisfied		−0.395	−0.575	2.304	7.573	0.129	0.006	0.673	0.563

**Table 6:** Two types of consumers regulatory satisfaction model parameter estimation results.

Variables	Ref to	*β*		Wald		*p* value		OR Value	
		Direct Experience	Indirect Experience	Direct Experience	Indirect Experience	Direct Experience	Indirect Experience	Direct Experience	Indirect Experience
**Knowledge of food safety**	Very satisfied							1.000	1.000
Very dissatisfied		−1.528	−1.311	17.768	13.122	0.000	0.000	0.217	0.270
Not very satisfied		−0.833	−1.152	10.263	25.691	0.001	0.000	0.435	0.316
General		−0.673	−0.827	8.009	16.568	0.005	0.000	0.510	0.437
Satisfied		−0.307	−0.262	1.802	1.828	0.179	0.176	0.736	0.769
Local food confidence level	Very satisfied							1.000	1.000
Very dissatisfied		−2.311	−3.026	17.322	30.880	0.000	0.000	0.099	0.048
Not very satisfied		−2.092	−2.320	46.422	89.752	0.000	0.000	0.123	0.098
General		−1.607	−1.629	33.017	59.722	0.000	0.000	0.200	0.196
Satisfied		−0.736	−0.824	7.935	18.534	0.005	0.000	0.479	0.439
Food safety regulatory satisfaction	Very satisfied							1.000	1.000
Very dissatisfied		−5.038	−4.257	96.219	82.316	0.000	0.000	0.006	0.014
Not very satisfied		−3.839	−3.264	141.984	173.373	0.000	0.000	0.022	0.038
General		−2.776	−2.446	93.418	134.213	0.000	0.000	0.062	0.087
Satisfied		−1.667	−1.597	39.625	69.845	0.000	0.000	0.189	0.202
